# Chloroplast-Expressed MSI-99 in Tobacco Improves Disease Resistance and Displays Inhibitory Effect against Rice Blast Fungus

**DOI:** 10.3390/ijms16034628

**Published:** 2015-03-02

**Authors:** Yun-Peng Wang, Zheng-Yi Wei, Yu-Ying Zhang, Chun-Jing Lin, Xiao-Fang Zhong, Yue-Lin Wang, Jing-Yong Ma, Jian Ma, Shao-Chen Xing

**Affiliations:** 1 Faculty of Agronomy, Jilin Agricultural University, No. 2888 Xincheng Street, Changchun 130118, China; E-Mails: wangypbio@163.com (Y.-P.W.); 99n2@163.com (J.-Y.M.); 2 Agro-Biotechnology Research Institute, Jilin Academy of Agricultural Sciences, No. 1363 Shengtai Street, Changchun 130033, China; E-Mails: weizy@cjaas.com (Z.-Y.W.); yuying0609@126.com (Y.-Y.Z.); lincj@cjaas.com (C.-J.L.); zhongxf@cjaas.com (X.-F.Z.); wangyuelin1989@163.com (Y.-L.W.); 3 College of Biological Sciences, China Agricultural University, No. 2 West Yuanmingyuan Road, Beijing 100094, China

**Keywords:** antimicrobial peptide, chloroplast transformation, rice blast, tobacco

## Abstract

Rice blast is a major destructive fungal disease that poses a serious threat to rice production and the improvement of blast resistance is critical to rice breeding. The antimicrobial peptide MSI-99 has been suggested as an antimicrobial peptide conferring resistance to bacterial and fungal diseases. Here, a vector harboring the *MSI-99* gene was constructed and introduced into the tobacco chloroplast genome via particle bombardment. Transformed plants were obtained and verified to be homoplastomic by PCR and Southern hybridization. In planta assays demonstrated that the transgenic tobacco plants displayed an enhanced resistance to the fungal disease. The evaluation of the antimicrobial activity revealed that the crude protein extracts from the transgenic plants manifested an antimicrobial activity against *E. coli*, even after incubation at 120 °C for 20 min, indicating significant heat stability of MSI-99. More importantly, the MSI-99-containing protein extracts were firstly proved *in vitro* and *in vivo* to display significant suppressive effects on two rice blast isolates. These findings provide a strong basis for the development of new biopesticides to combat rice blast.

## 1. Introduction

Rice blast is one of the three major fungal diseases causing economic losses in rice production of approximately US $60 million every year [1]. The physiological races of rice blast pathogens vary from year to year due to many factors such as weather, strains and cultivation habits. Therefore, it is difficult to obtain a rice cultivar displaying resistance to multiple races of rice blast pathogens using the normal breeding methods. Indeed, a rice cultivar resistant to the predominant race of pathogens could lose resistance just a few years after its release [2]. It has been reported that transgenic rice expressing chitinase [3], chitosan [4], and resistance genes [5] can improve resistance against rice blast, but there is no disease-resistant transgenic rice cultivar in commercial production. In practice, phytodrugs such as tricyclazole are commonly used to fight rice blast.

Antimicrobial peptides are generally composed of 12–50 amino acid moieties. The mechanism underlying the antimicrobial activity is mainly attributed to disrupting the plasma membrane, leading to lysis of the cell and eventually the cell death [6]. Due to their indirect and non-specific resistance mechanisms, it is difficult for pathogens to develop resistance towards antimicrobial peptides; Therefore, they present greater advantages over the insecticides and antimicrobial pesticides commonly applied in the food industry and modern agriculture, as they are environmentally friendlier [7].

The 23-amino-acid long antimicrobial peptide MSI-99 is a derivative of Magainin II, with a His7 to Lys substitution that prevents the gene from recognition and digestion by plant endonucleases, while maintaining its normal functions. Therefore, it can be applied to multiple explants for genetic transformation, with a potential for improving crop disease resistance. Up to now, MSI-99 and Myp20 are the only two antimicrobial peptides among magainins and derivatives that have been successfully expressed in transgenic crops among magainins and derivatives [8]. To improve crop resistance against various bacterial or fungal pathogens, the *MSI-99* gene has been introduced into multiple plants, including tobacco [9], banana [10], tomato [11], potato [12] and cole [13]. For instance, upon high MSI-99 expression in tobacco chloroplasts, the leaf extracts from transgenic plants inhibited the growth of over 95% pregerminated spores of three fungal pathogens, including *Aspergillus flavus*, *Fusarium moniliforme* and *Verticillium dahlia* [9].

Chloroplast transformation provides numerous advantages over conventional nuclear transformation [14,15,16,17], especially, the high expression level of the foreign protein that can be obtained [18,19,20]. This study aimed to express the antimicrobial peptide in tobacco chloroplasts and to assess the inhibitory effects of plant-produced MSI-99 on rice blast fungus isolates. Total protein samples obtained from the leaves of transplastomic tobacco plants were assessed for their inhibitory effects on the mycelial growth of two rice blast isolates. Our results described herein demonstrate that tobacco chloroplast-derived MSI-99 exhibit significant inhibitory effects on physiological strains of rice blast. These findings provide a strong basis for the breeding of new, resistant to rice blast, chloroplast transgenic rice cultivars, and establish the foundation for the development of novel biopesticides.

## 2. Results and Discussion

### 2.1. Construction of the Expression Vector for Tobacco Chloroplast Transformation

**Figure 1:**
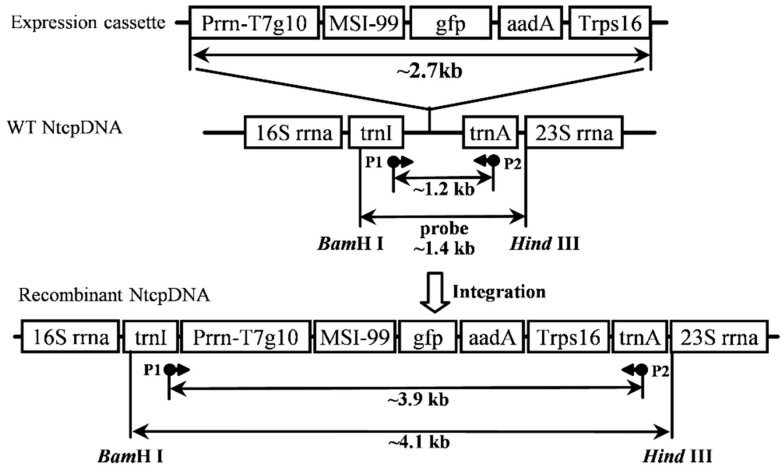
Expression vector construction. The expression vector contained the Prrn-T7g10-MSI99-gfp-aadA-Trps16 expression cassette which was inserted into the Nsi I site between trn I and trn A on the wild-type tobacco plastid genome. P1 and P2 were the primers used for PCR. Two bands of ~1.2 and ~3.9 kb were amplified from the wild-type plants and the transgenic plants, respectively; BamH I and Hind III were used for digestion in Southern blot hybridization. The ~1.4 kb fragment between trnI and trnA represented the probe.

The tobacco chloroplast transformation vector was constructed as shown in Figure 1. This vector contained 16*S*-*trn*I and *trn*A*-*23*S* as flanking homologous sequences, between which the chemically synthesized cassette was inserted into the *Nsi* I site between *trn* I and *trn* A genes. The Prrn promoter and Trps16 terminator were the regulatory elements from the tobacco chloroplast genome (accession number: NC_001879).

### 2.2. Selection of Homoplastomic Plants, Molecular Testing and Antibiotic Resistance of T_1_ Offspring

**Figure 2:**
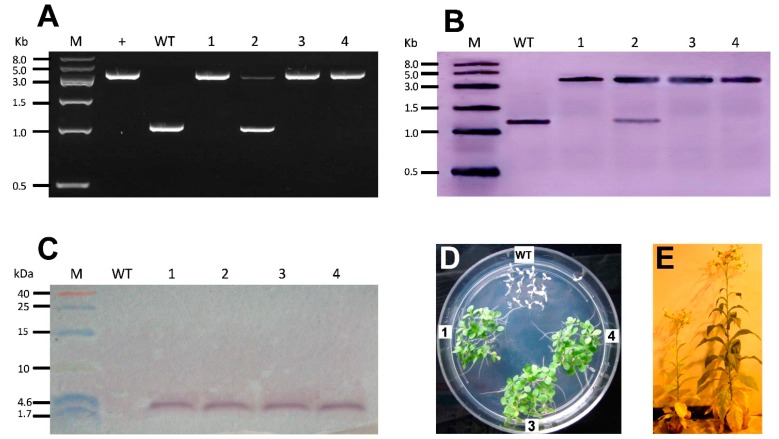
Achievement of homoplastomic plants, molecular testing and resistance of T_1_ progenies. Putative transgenic plants were verified by PCR (**A**) and Southern blot hybridization (**B**), indicating the homoplastomic status of lines 1, 3 and 4, but the heteroplastomic status of line 2; (**C**) Western blot analysis for MSI-99 expression. Lines 1 to 4 showed protein detection in the four transgenic lines but not in the WT control. M, DNA marker; +, plasmid positive control; WT, wild-type control; 1–4, transgenic plants; (**D**) Homoplastomic T_1_ progeny on MS media with 500 mg/L spectinomycin. WT indicates wild-type seedlings, while 1, 3 and 4 indicate transgenic seedlings; and (**E**) Soil-grown homoplastomic plant (**left**) showed growth retardation compared with wild type plant (**right**).

The vector was introduced into tobacco chloroplasts using particle bombardment. After three rounds of antibiotic selection, four regenerated plants were obtained, and three of them were confirmed to be homoplastomic by the primers P1/P2 (Figure 2A). According to the mechanism of homologous recombination, it was predicted that only a ~1.2 kb fragment could be amplified from wild-type plants, while a ~3.9 kb fragment from transgenic plants was anticipated because of the insertion of the expression cassette. Therefore, both fragments could be amplified in heteroplastomic plants, whereas only the ~3.9 kb fragment would be observed in homoplastomic plants (Figure 2A). As illustrated in Figure 2A, three lines (1, 3 and 4) were homoplastomic, whereas line 2 was heteroplastomic.

To validate these findings, Southern blot hybridization was conducted, and the result is depicted in Figure 2B. As expected, the wild-type plant displayed a ~1.4 kb signal, the homoplastomic plant yielded a signal of ~4.1 kb, and the heteroplastomic plant had signals at both positions. This further confirmed that lines 1, 3 and 4 were homoplastomic, while the line 2 was heteroplasmic, in agreement with the PCR results.

With regard to the expression of MSI-99, a Western blot was performed using a multiclonal antibody directed against MSI-99. As shown in Figure 2C, in the four transplastomic plants, the antibody detected the expected ~2.7 kDa protein, whereas there was no band in non-transformed control plant, indicating the effective expression of MSI-99 in transgenic plants, regardless of whether the plants are homoplastomic or not.

After strict selfing, the T_1_ offspring of three homoplastomic lines grew normally on Murashige and Skoog (MS) media containing 500 mg/L spectinomycin as shown in Figure 2D, revealing the expression and stable inheritance of the *aadA* gene, which confers the resistance to spectinomycin. In contrast, the leaves of wild-type plants were bleached. Unfortunately, under the normal growth conditions, in which the control plants were grown, the transgenic plants manifested growth retardation (Figure 2E), although they also could finally blossom and fruit.

### 2.3. Quantification of MSI-99 Expression in T_1_ Generation

**Figure 3:**
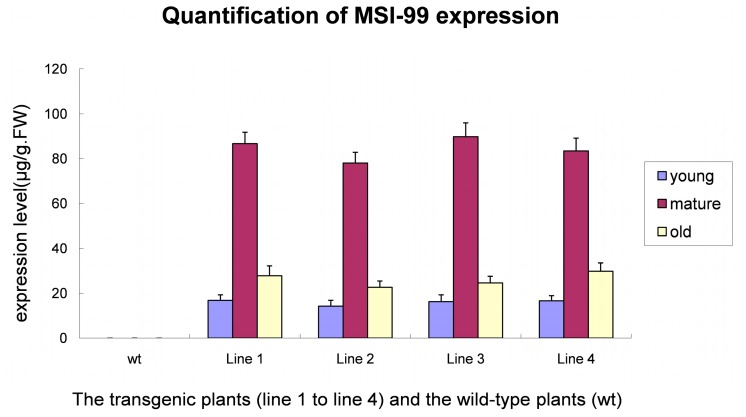
Quantification of MSI-99 expression levels in T_1_ generation by ELISA. Four transplastomic lines (line 1 to line 4) were used to determine separately the expression of MSI-99 in young, mature and old leaves. The wild type plant (wt) was set as the control.

Quantification of MSI-99 was done by the enzyme-linked immunosorbent assay (ELISA), the results of which are presented in Figure 3. It is obvious that the expression level displayed in the mature leaves of four lines were exceedingly higher than that in the young and old leaves, whereas the expression of MSI-99 in the wild-type plants was not detectable. The highest expression level of MSI-99 in the mature leaves of line 3 reached up to 89.75 µg/g·FW (fresh weight). In all four transgenic lines, the old leaves demonstrated relatively higher MSI-99 expression than the young leaves.

### 2.4. In Vivo Assessment of Green Fluorescent Protein (GFP) Expression

**Figure 4:**
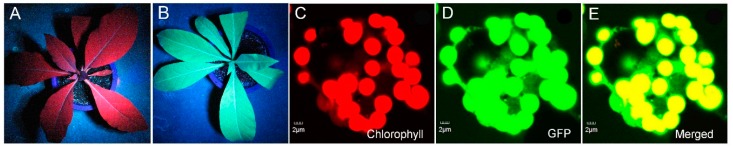
Assessment of GFP expression. (**A**) Under UV light, the non-transgenic plant appeared red due to chlorophyll auto-fluorescence; (**B**) The transgenic plant appeared green due to GFP fluorescence. Using confocal microscopy, the protoplasts isolated from a leaf of a transgenic plant appeared red due to chlorophyll auto-fluorescence (**C**) and green due to GFP expression (**D**); and (**E**) Represents a merged image of (**C**,**D**).

As shown in Figure 4, under UV light, the transgenic plants displayed green fluorescence in the dark (Figure 4B), whereas only red auto-fluorescence was observed in the control plants (Figure 4A). Moreover, through the examination of leaf protoplasts from the transgenic plants via confocal microscopy, strong green fluorescence emitted by protoplasts was detected (Figure 4D), suggesting high expression of GFP.

### 2.5. Resistance to the Fungal Disease Caused by Alternaria alternata and in Vitro Antifungal Activity of MSI-99 against Rice Blast Pathogens

**Figure 5:**
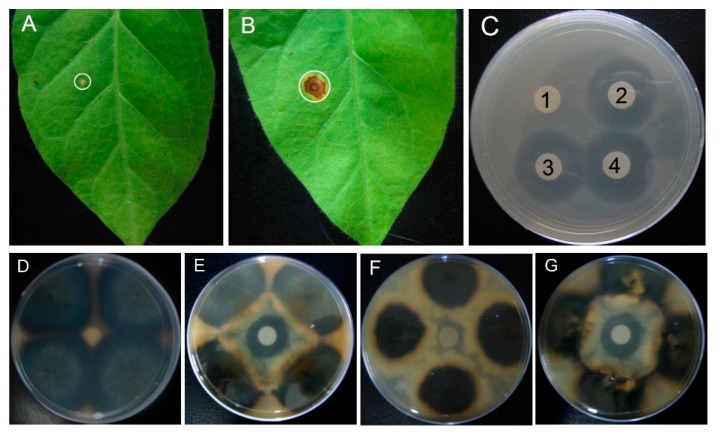
*In planta* assay of resistance to *Alternaria alternata* and *in vivo* evaluation of MSI-99 antifungal activity. (**A**) No necrotic lesions were formed on the leaves of the transgenic plants inoculated with the fungus *Alternaria alternata* whereas obvious disease symptoms were observed on the leaves of the control plants (**B**); (**C**) Inhibitory effect of the MSI-99-containing protein on *E. coli.* The wild-type plant extracts showed no inhibitory zone (C1), while the crude extracts from transgenic plants formed inhibitory zones of similar sizes whether untreated (C2), incubated at room temperature (C3) or held at 120 °C for 20 min (C4); The crude extracts from the wild-type plants displayed no effect on the mycelial growth of rice blast pathogen isolates ZA11 (**D**) and ZE18 (**F**); In contrast, the transgenic plants extracts inhibited the mycelial growth of isolates ZA11 (**E**) and ZE18 (**G**), forming overt inhibitory zones.

Three homoplastomic plants were inoculated with the causative agent of the fungal disease, *Alternaria alternate*, and the results evidenced no necrotic lesions formed in the leaves of transgenic plants (Figure 5A), whereas the non-transformed controls displayed necrotic symptoms (Figure 5B). To evaluate the antimicrobial activity of MSI-99, we first employed *E. coli* to validate whether MSI-99 had the expected antibacterial effect. The control, the wild-type plant extract, exhibited no antibacterial activity, which was concluded due to the lack of an inhibitory zone (Figure 5C1). The MSI-99-containing plant extracts, however, placed at room temperature for 20 min showed antibacterial activity (Figure 5C3), similar to that of the untreated extracts (Figure 5C2), forming an inhibitory zone. Importantly, when the plant extracts were heated at 120 °C for 20 min, they still retained their antibacterial activity: the size of the inhibitory zone (Figure 5C4) was similar to that of the unheated extract, indicating the considerable heat stability of this protein. More importantly, MSI-99 manifested distinct inhibitory effects against the two rice blast isolates. The protein extracts from the wild-type control had no inhibitory influence on the mycelial growth of ZA11 (Figure 5D) and ZE18 (Figure 5F), as no inhibitory zone was formed. In contrast, the protein extracts from transgenic plants exerted a significant suppressive effect on the mycelial growth of ZA11 (Figure 5E) and ZE18 (Figure 5G). The growth of mycelia was greatly inhibited before approaching the filter paper and the inhibitory zones were clearly observed. To our knowledge, this is the first report suggesting the inhibitory effect of MSI-99 on rice blast pathogens. These findings provided a basis for the development of new biopesticides to combat rice blast.

### 2.6. In Plant Resistance to Rice Blast Magnaporthe oryzae

**Figure 6:**
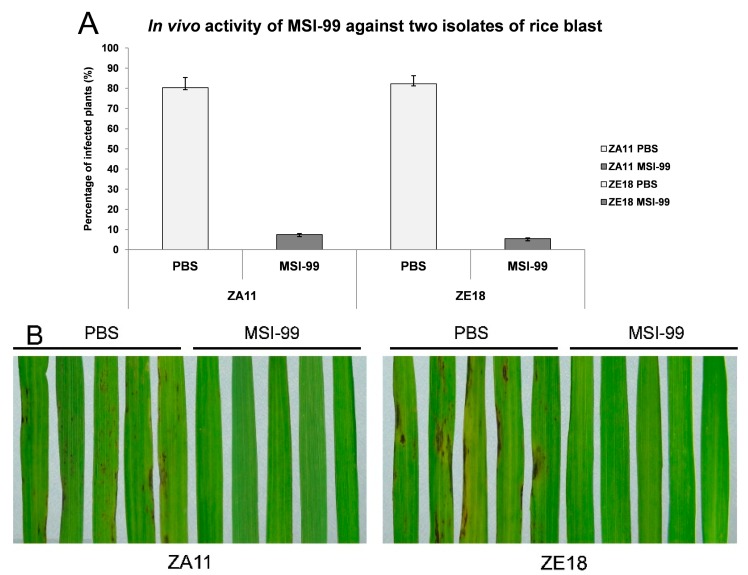
In Planta resistance to two isolates of rice blast ZA11 and ZE18. (**A**) *In vivo* activity of MSI-99 against ZA11 and ZE18. The percentage of infected plants was calculated after the seedlings inoculated with ZA11 and ZE18 were sprayed twice with MSI-99-containing crudes and PBS buffer; (**B**) Disease reaction of three-week-old plants to two rice blast isolates, ZA11 and ZE18. Lesions were observed on leaf sections sprayed with PBS buffer, and no disease phenotypes were recorded when sprayed with MSI-99-containing crudes. Photos were taken 7 days after spray inoculation.

Firstly, the minimum inhibitory concentration (MIC) of MSI-99 for ZA11 and ZE18 was determined to be 0.625 and 1.25 μg/mL, respectively, and then the antifungal activity of MSI-99-containing crudes was tested against ZA11 and ZE18. The results demonstrated the seedlings of rice variety Lijiang Tuan Heigu (LTH) can be protected effectively from infection of two isolates. The percentage of infected plants with ZA11 and ZE18 is 7.55% and 5.49%, respectively, after sprayed with MSI-99-containing crudes. Meanwhile, the control plants sprayed with phosphate buffer saline (PBS) butter showed much higher ratio of infection, 80.32% for ZA11 and 82.20% for ZE18 (Figure 6A). The performance of representative leaf sections from indicated plants is in agreement with the statistical data. The disease lesions were clearly observed on the leaf sections when the plants were sprayed with PBS buffer after inoculated with ZA11 and ZE18, but there are no necrotic symptoms on the leaf sections when sprayed with MSI-99-containing crudes (Figure 6B), indicating the activity of rice blast is restrained by MSI-99.

Since the discovery of the first antimicrobial peptides, the cecropins, in the early 1980s, numerous representatives of this type have been described [21]. Over the past decades, studies have repeatedly confirmed that, to different extent, antimicrobial peptides have broad-spectrum activities against bacteria, fungi, viruses, protozoan and even cancer cells [22]. Given the misuse of phytodrugs and insecticides in rice production that increases the pathogen resistance, the application of antimicrobial peptides as potential substitutes offers an important opportunity.

Our study is the first to report the lethal effect of MSI-99 on two isolates of rice blast pathogens which constitute the dominant population in Northeast China. The results of *in vivo* assays especially provide us an alternative to protect rice form blast infection. Rice production in this region could benefit from transgenic rice cultivars, once achieved through genetic manipulations such as nuclear or plastid transformation. Additionally, by using modified gel, we also detected directly the existence of MSI-99 in the transplastomic leaves, whereas DeGray *et al.* [9] at the time of their investigations were unable to identify it due to the small molecule size of 2.7 kDa. As mentioned above, the chloroplast-produced MSI-99 is capable of inhibiting the growth of pregerminated spores of three tobacco fungal pathogens [9]. Similarly, our experiment confirmed the resistance of MSI-99 to another different fungal pathogen, extending the range of resistance. MSI-99 could also be obtained from the transplastomic plants and purified as a biopesticide for the prevention of rice blast. Moreover, studies have indicated that antimicrobial peptides generally tolerate high temperatures, e.g., 100 °C for 10–15 min [23,24]. We found that MSI-99 retains its activity at 120 °C for at least 20 min, which contributes to the overall improvement of the applicability and prolongs the efficacy of biopesticides.

A number of reports have suggested that chloroplast transformation exerts less negative influence on plant development compared with nuclear transformation [20,25,26]. However, excessively high expression can affect the normal growth of the plant. For instance, Oey *et al.* [27] found that the overexpression of the exogenous lytic enzyme PlyGBS significantly affected tobacco growth. In addition, Petersen and Bock [28] overexpressed the genes encoding cell wall-degrading enzymes from *Thermobifida fusca* in tobacco and found that the transgenic offspring showed severe pigment-deficient phenotypes. We demonstrated that the expression of MSI-99 in tobacco inhibits the normal growth of the plant, yielding smaller individuals compared with the wild-type control (Figure 2E), although they still bloomed and fruited. These findings are in contrast with a previous report that MSI-99 transgenic tobacco plants grow normally [9]. This was probably caused by the overexpression of MSI-99 and GFP, resulting in a heavy burden on plant metabolism.

In general, prokaryotic organisms like *E. coli* are used as a platform to express functionally important proteins for practical applications. Due to its antimicrobial activity, MSI-99 cannot be produced in prokaryotic systems. Therefore, it is more convenient to take advantage of the high expression of the target protein in chloroplasts for the improvement of crop resistance. Meanwhile, given the possibility that the overexpression of a target protein may negatively affect the growth of transgenic plants [14,29], a balance between the expression level and the growth should be considered when designing expression vectors, instead of over-pursuing high expression levels at the cost of the overall plant growth.

## 3. Experimental Section

### 3.1. Plant Materials and Growth Conditions

Tobacco (*Nicotiana alata* Link et Otto cv. Grandiflora) seeds were surface-sterilized and grown on 1/2 MS medium supplemented with 8 g/L agar and 30 g/L sucrose under a photoperiod of 16-h light/8-h dark. The leaves were used for DNA extraction, genetic transformation and disease resistance assays.

The rice (*Oryza sativa*) susceptible variety Lijiang Tuan Heigu (LTH) were grown in a growth chamber at 28 °C and 75% relative humidity under a photoperiod of 14-h light/10-h dark. Three-week-old seedlings were designed statistically with three replicates pretreatment and prepared for spray inoculation.

### 3.2. Construction of the Tobacco Chloroplast Transformation Vector

The transformation vector was constructed following the protocol described previously in our lab [30] with modification. Briefly, based on the genomic sequence of the tobacco chloroplast genome (accession number: NC_001879), two primers, Pa (5'-ACAGAGGATGCAAGCGTTAT-3') and Pb (5'-CACTGAGCGATCATTTAGGG-3') were designed. The 16S-trnI-trnA-23S fragment in the inverted repeat (IR) region of the chloroplast genome was amplified by PCR as a homologous sequence to construct the expression vector. The PCR program was set as follows: 95 °C pre-denaturation for 5 min; 95 °C denaturation for 45 s, 50 °C annealing for 40 s, 72 °C elongation for 2 min, 30 cycles in total; 72 °C post-elongation for 5 min. The PCR products were ligated into the pEASY-Blunt Simple vector and sequenced. The vector containing the correct insert was digested by *Nsi* I endonuclease, and then the chemically synthesized expression cassette Prrn-T7g10-MSI99-gfp-aadA-Trps16 was inserted to produce a tobacco-specific expression vector (Figure 1).

### 3.3. Chloroplast Transformation and Homoplastomic Selection

Chloroplast transformation was performed as described previously [30], using a PDS 1000/He gene gun. The bombarded leaves were placed abaxial side up on the recovery MS media (4.4 g/L MS salt, 30 g/L sucrose and 8 g/L agar, pH 5.8) and cultured for 3 days at 25 °C in the dark. Then, the leaves were cut into small pieces (5 mm × 5 mm) and placed abaxial side down on the selection medium (MS salt, 30 g/L sucrose, 2 mg/L 6-benzylaminopurine (BA), 0.1 mg/L naphthaleneacetic acid (NAA), 500 mg/L spectinomycin and 8 g/L agar, pH 5.8) at 25 °C under a 16/8 h light/dark cycle. The selection media were replaced every 20 days until completion of the first round of selection when adventitious buds appeared. When the adventitious buds reached 3 cm, they were examined by PCR amplification. The leaves of the PCR-positive buds were submitted to a second and third round of regeneration under selection, as described above, until confirmation of the homoplastomic adventitious buds by PCR assays. The homoplastomic buds were transferred onto a rooting medium (MS salt, 30 g/L sucrose, 500 mg/L spectinomycin, and 8 g/L agar, pH 5.8). After hardening and transplanting, homoplastomic tobacco plants were obtained and reconfirmed by Southern blot analysis. After strict selfing, the T_1_ seeds were collected and sterilized by 10% (*v*/*v*) perhydrol for germination on wet filter paper. The T_1_ seedlings were transferred onto a MS medium containing 500 mg/L spectinomycin for a week.

### 3.4. PCR Amplification

The total DNA was extracted according to previous protocols [31] for PCR and Southern blot analysis. The positions of primers P1 (5'-GGTCGGAACAAGTTGATAG-3') and P2 (5'-CAGTAGAGTCTTTCAGTGGC-3') in the tobacco chloroplast genome are indicated in Figure 1. The following PCR program was used to amplify this region: 95 °C pre-denaturation for 5 min; 95 °C denaturation for 20 s, 56 °C annealing for 20 s, 72 °C elongation for 2 min, 30 cycles in total; 72 °C post-elongation for 5 min. PCR products were separated on 0.8% (*w*/*v*) agarose gel.

### 3.5. Southern Blot Analysis

A total of 4 μg of total DNA was digested using *Bam*H I and *Hind* III. The digestion mixture was dissolved on 0.8% (*w*/*v*) agarose gel and transferred onto a nitrocellulose membrane. Southern blot analysis was performed according to the kit instructions (Roche, Basel, Switzerland). The probe shown in Figure 1 represents a ~1.4 kb fragment.

### 3.6. Tricine-SDS-PAGE, Western Blot Analysis and ELISA Assay

Crude protein from mature leaves of four transgenic plants was extracted and separated by Tricine-SDS–PAGE on a 16.5% (*w*/*v*) gel. For the Western blot, the proteins were transferred after migration in a Tricine-polyacrylamide gel to a nitrocellulose membrane (0.22 μm, Millipore, Billerica, MA, USA) using a Trans-Blot Semi-Dry electrophoretic Transfer Cell (200 mA, 30 min, Bio-Rad, Hercules, CA, USA). The nitrocellulose membrane was then saturated with 5% BSA (*w*/*v*) in TBS-Tween [25 mM Tris-HCl pH 7.6, 0.15 M NaCl, and 0.05% (w/v) Tween-20]. Incubation was performed with the primary antibody directed against the antimicrobial peptide MSI-99, prior to the incubation with the secondary antibody (1:3000, anti-mouse IgG alkaline phosphatase conjugate, Novagen, Darmstadt, Germany). The labeled proteins were visualized by adding a substrate BCIP/NBT (Roche).

The quantification of MSI-99 in the transgenic plant crude extract was carried out using ELISA (Invitrogen, Waltham, MA, USA). Approximately 100 mg leaf samples of four transgenic lines, together with the wild-type plant, were collected separately from young, mature and old leaves, and grounded in liquid nitrogen for crude protein extraction. The ELISA was performed according to the manufacturer’s manual.

### 3.7. Assessment of GFP Expression

Soil-grown homoplastomic plants, together with non-transgenic controls were submitted to ultra-violet (365 nm) irradiation in the dark and photographs were taken. Protoplasts were isolated from the transgenic leaves for investigation by confocal laser microscopy. Leaves with veins removed were treated with 13% (*w*/*v*) mannitol for 1 h and transferred into CPW buffer (pH 5.8) containing 1.0% (*w*/*v*) cellulose, 0.2% (*w*/*v*) pectinase, and 6% (*w*/*v*) mannitol. The samples were then placed on a rocker at 60 rpm for 12 h, at 28 °C, in the dark. The isolated protoplasts were placed on coverslips and examined under 1000× magnification and 488 nm laser scanning on an Olympus FV1000-IX81 confocal laser microscope (Tokyo, Japan) for GFP expression evaluation.

### 3.8. In Planta Assay for Fungal Disease Resistance in Transgenic Tobacco and in Vitro Evaluation of Antimicrobial Activity against the Growth of E. coli

Conidiospores of *Alternaria alternata* were collected from potato dextrose agar (PDA, 200 g/L potato, 20 g/L sucrose, and 8 g/L agar, pH 7.0) media by washing a 7-day-old culture with a solution of 0.5% (*w*/*v*) Tween-20 and rubbing the surface. The spore suspension was adjusted to a final inoculum density of 10^7^ spores/mL. The homoplastomic plants expressing-MIS-99 were inoculated according to the published protocols [32]. Leaves of the wild-type plants were also inoculated as controls. Photos were taken 10 days after the inoculation. The antimicrobial activity of MSI-99 was assessed in the total protein extracts which were extracted from mature leaves of both homoplastomic and non-transgenic plants, and adjusted to 5 mg/mL in PBS. Sterilized 5 mm filter paper discs were soaked in crude protein extracts for 5 min and grouped into three treatments: untreated, treated at room temperature, and treated at 120 °C for 20 min. After the treatment, the discs were placed on Luria-Bertani (LB) plates containing *E. coli* grown overnight at 37 °C, and the growth inhibition zone sizes were examined after an additional incubation day.

### 3.9. In Vitro and in Vivo Bioassays of Antifungal Activity of MSI-99

Two rice blast isolates ZA11 and ZE18, which are dominant in Northeast China rice growing region, were collected from Agriculture Culture Collection of China (ACCC) and used to evaluate the antifungal activity of MSI-99. The minimum inhibitory concentration (MIC) was determined in 96-well plates by using dimethylthiazol (MTT) staining. For the *in vitro* experiment, the fungi were cultured on plates of PDA (pH 7.0) for a week. Then, sterilized filter paper pieces soaked with crude protein extracts from transgenic plants were placed in the middle of the each plate, surrounded evenly by four fungal lawns of 5 mm diameter. Control plates contained discs soaked with diluted non-transgenic plants extracts. Finally, the plates were inverted and cultured for 10–15 days at 28 °C to assess the inhibition zone sizes.

For the *in vivo* assays, the inoculum concentration of each strain was adjusted to 5 × 10^5^ spores/mL for spray inoculation. After inoculation, the seedlings were grown for 24 h at 28 °C and 95% relative humidity in the dark. The crud protein extracts diluted with PBS to 8.0 μg/mL of MSI-99 were sprayed for the first time, and the spraying was repeated once after 48 h. The volume of spraying is 3 mL per ten seedlings. The disease symptoms were investigated 7 days after inoculation as described previously [33] which the plants rated 0–3 were considered resistant and those rated 4–9 were classified into susceptible. In contrast, the seedlings sprayed with PBS were served as control.

## 4. Conclusions

Homoplastomic tobacco plants were successfully obtained and the MSI-99-containing crude proteins showed an inhibitory effect on the mycelial growth of rice blast pathogens, forming distinct inhibitory zones in the plates. In planta assays showed that the expression of MS-I99 in the transgenic tobacco plants enhanced the resistance to another fungal disease, caused by *Alternaria alternate*. Moreover, the two isolates spray-inoculated on the susceptible plants can effectively be restrained by MSI-99. Additionally, MSI-99 is able to preserve its inhibitory activity even after the treatment with 120 °C for 20 min, indicating significant heat stability.
